# Meta-analysis of brucellosis and relative risks

**DOI:** 10.1097/MD.0000000000047696

**Published:** 2026-02-28

**Authors:** Lei Feng, Yi Shang

**Affiliations:** a Shangrao Center for Disease Control and Prevention, Jiangxi, China; b Ganzhou Center for Disease Control and Prevention (Municipal Health Comprehensive Supervision and Law Enforcement Bureau), Jiangxi, China.

**Keywords:** brucellosis, dietary habits, meta-analysis, risk factor

## Abstract

**Objective:**

A meta-analysis was used to study the main risk factors of human brucellosis, thereby providing references for formulating brucellosis prevention and control strategies.

**Methods:**

Databases including PubMed, Web of Science, China National Knowledge Infrastructure, Wanfang Data were systematically searched to identify relevant studies on dietary risk factors for human brucellosis worldwide, and the studies were screened according to the inclusion criteria, and a total of 57 studies were included. Seven dietary-related risk factors were identified: animal milk consumption, dairy consumption, raw or undercooked meat consumption, sick or dead animal meat consumption, blood consumption, zoonotic drinking water sources, and poor hand hygiene before meals. Meta-analysis was conducted using Review Manager 5.3 software (The Nordic Cochrane Centre), and either the fixed-effects model or random-effects model was applied for meta-analysis to comprehensively quantitatively assess the risk factors of brucellosis spread in dietary habits. Additionally, sensitivity analyses and test for publication bias were performed to ensure the robustness of the findings.

**Results:**

Meta-analysis of 57 articles included in the study showed that animal milk consumption (odds ratio [OR] = 5.12, 95% confidence interval [CI]: 3.56–7.37), dairy consumption (OR = 2.28, 95% CI: 1.31–3.95), sick or dead animal meat consumption (OR = 4.54, 95% CI: 3.18–6.48), blood consumption (OR = 2.72, 95% CI: 1.83–4.04) were all risk factors for the transmission of human brucellosis in terms of dietary habits.

**Conclusion:**

Animal milk consumption, dairy consumption, sick or dead animal meat consumption, and blood consumption are the main risk factors of brucellosis.

## 1. Introduction

Brucellosis, also known as “undulant fever,” commonly known as “lazy man’s disease,” is a critical zoonotic disease caused by Brucella infection. Its typical clinical manifestations include chills, fever, fatigue, hyperhidrosis, and musculoskeletal pain, with potential complications such as meningitis and osteoarthritis in severe cases, which may even be life-threatening.^[1]^ Globally, brucellosis is widely prevalent, especially in the Mediterranean region, Latin America, Africa, and Asia.^[2]^ The latest research shows that the global incidence of brucellosis is increasing year by year, with about 2 million new human cases each year.^[3]^ In China, brucellosis ranks among the top 5 reported cases of class B infectious diseases, mainly concentrated in some areas with developed animal husbandry, such as Xinjiang, Inner Mongolia, Tibet, and Gansu in recent years.^[4]^

The infection risk routes of brucellosis are mainly through contact transmission, respiratory transmission and digestive tract transmission. Although people are rarely able to get sick through direct contact with sick animals in their daily lives, it is common through foodborne infections, such as ingestion of animal-derived foods contaminated with Brucella and not thoroughly sterilized, such as raw milk, undercooked dairy products (such as fresh cheese), or undercooked meat.^[5]^ Understanding the risk factors of brucellosis in dietary habits to improve people’s awareness of brucellosis can help reduce the incidence of brucellosis in people.^[6]^ However, existing studies on dietary risk factors are scattered across different regions, and a comprehensive synthesis of global evidence is lacking. This highlights the need for a systematic analysis to clarify the association between dietary patterns and brucellosis, thereby providing targeted support for prevention and control efforts.

To address this research gap, the present study employs a meta-analysis approach to systematically synthesize relevant studies from 25 countries and regions worldwide. The objective is to explore the association between different dietary patterns and the occurrence of brucellosis, thereby providing scientific evidence to support strategies for reducing incidence and blocking transmission pathways.

## 2. Materials and methods

### 2.1. Source of data

This study searched Chinese and English studies on brucellosis and dietary habits from academic databases including Web of Science, PubMed, China National Knowledge Infrastructure, and Wanfang Data. English search terms are “Brucella infection,” “Brucellosis,” “Malta fever,” “Undulant fever,” “Rock Fever,” “Cyprus fever,” “Gibraltar fever,” “Risk factor,” “Hazards,” “Dietary factors,” “Dietary,” “Risk score,” “Risk”; Chinese search terms included “brucellosis,” “brucella,” “Undulant fever,” “Malta fever,” Mediterranean flaccid fever, risk, diet, factor, etc. This meta-analysis is a secondary analysis based on publicly available data and does not involve new study subjects and therefore does not require ethics committee approval.

### 2.2. Document entry and arrangement standard

Inclusion criteria: the search included all studies published domestically and internationally since the database establishment, and the research topic should include the relationship between brucellosis and dietary factors; studies designed as case-control or cross-sectional studies; and the total number of people in each group needs to be reported in the literature, or the above indicators can be calculated from the existing data.

Exclusion criteria: exclude brucellosis-related studies that only focused on nonhuman hosts such as cattle, sheep, dogs, etc as research subjects; exclude reviews, experimental papers, conference papers, lectures, meta-analysis, etc; eliminate studies with incomplete data; exclude the literature whose quality evaluation score does not reach 4 points; and exclude literature that does not involve dietary factors.

### 2.3. Literature screening and quality evaluation

The process of literature screening is as follows: 2 experienced researchers screen and evaluate the quality of the literature according to the preset admission criteria. First of all, the title and abstract of the literature are carefully read to carry out preliminary screening; subsequently, for the literature that met the requirements of the initial screening, the full text was further read and the key data were extracted. The extracted data are as follows: animal milk consumption; dairy consumption; raw or undercooked meat consumption; sick or dead animal meat consumption; blood consumption; zoonotic drinking water sources; and poor hand hygiene before meals, and the articles with extractable data information are included in the study. Quality evaluation: In this meta-analysis, the included studies will be evaluated for scientifically rigorous quality. The 2 researchers uniformly used the Newcastle-Ottawa Scale to independently score the quality of the studies, and if they encountered differences, they were included in a third person for discussion and analysis. The assessment dimensions included selection, comparability, and exposure in case-control study or outcome in cross-sectional study. In this paper, the research quality is divided into 3 grades: 1 to 3 scores are defined as low quality research; 4 to 7 points are classified as medium quality research; a score of 8 to 9 is judged as high-quality research. After the rigorous evaluation of the literature quality, the effective information of the included literature is extracted: basic information: literature title, publication year, author name, research place, etc; study characteristics: sample size of case group and control group, study protocol design, study means to control deviation, etc; and results: data on the association between dietary factors and brucellosis prevalence.

### 2.4. Statistical analysis

Review Manager 5.3 software was used to conduct a meta-analysis of all final included studies.^[7]^ The odds ratio (OR) and its 95% confidence interval (CI) were used as indicators to evaluate the effect.^[8]^ Heterogeneity among studies was evaluated using the *Q*-test combined with *I*^2^ statistic. If *I*^2^ < 50%, it indicates low heterogeneity among studies. At this time, a fixed-effect model should be used to combine the effect sizes for meta-analysis. If *I*^2^ > 50%, it indicates that there is high heterogeneity among the study groups, and a random-effects model should be selected to combine effect sizes for meta-analysis. Finally, for the sake of scientific rigor, publication bias was evaluated by assessing the symmetry of funnel plots.

## 3. Result

### 3.1. Literature search status

Using the aforementioned search strategy, a total of 4179 Chinese and English studies were retrieved. After eliminating duplicates, 57 studies were finally included for subsequent meta-analysis. All studies are based on case-control or cross-sectional studies, focusing on the analysis of epidemiology and risk factors of brucellosis. The study included data from 25 countries and 10 provinces and municipalities directly under the Central Government in China, with a total of 3954 cases and 29,420 controls (Table 1).^[9-65]^

**Table 1:** General characteristics of included studies.

Included studies	Study place	Number		Study type	Risk factor
		Study group	Control group		
Chang^[9]^	China (Shanxi)	80	160	Case-control study	cdf
Cheng^[10]^	China (Liaoning)	98	1642	Cross-sectional study	d
Gao^[11]^	China (Hebei)	330	330	Case-control study	adg
Gao^[12]^	China (Hebei)	107	680	Case-control study	acdfg
Gao^[13]^	China (Hebei)	228	684	Case-control study	acdfg
Jiang^[14]^	China (Guizhou)	82	164	Case-control study	g
Liu^[15]^	China (Shandong)	81	162	Case-control study	ac
Man^[16]^	China (Heilongjiang)	30	90	Case-control study	a
Wang^[17]^	China (Shanxi)	54	292	Cross-sectional study	a
Xia^[18]^	China (Chongqing)	12	24	Cross-sectional study	d
Xue^[19]^	China (Qinghai)	141	6169	Cross-sectional study	ac
You^[20]^	China (Inner Mongolia)	6	494	Cross-sectional study	ac
Zhang^[21]^	China (Shandong)	32	87	Case-control study	adg
Zhang^[22]^	China (Jiangsu)	11	197	Cross-sectional study	g
Abbas^[23]^	Pakistan	21	286	Cross-sectional study	a
Abo-Shehada^[24]^	Jordan	56	247	Case-control study	ab
Etemadi^[25]^	Iran	141	156	Cross-sectional study	b
Al-Hakami^[26]^	Saudi Arabia	80	137	Cross-sectional study	ac
Ali^[27]^	Pakistan	20	439	Cross-sectional study	a
Ali^[28]^	Pakistan	18	196	Cross-sectional study	a
Almashhadany^[29]^	Iraq	40	284	Cross-sectional study	a
Al-Shaar^[30]^	Lebanon	50	95	Case-control study	bc
Asiimwe^[31]^	Uganda	45	90	Case-control study	ab
Aworh^[32]^	Nigeria	54	170	Cross-sectional study	abc
Cash-Goldwasser^[33]^	Tanzania	50	512	Cross-sectional study	ae
Cetinkaya^[34]^	Turkey	51	1001	Cross-sectional study	b
Charaa^[35]^	Tunisia	25	52	Case-control study	a
Earhart^[36]^	Uzbekistan	144	294	Case-control study	ab
Ejaz^[37]^	Pakistan	91	294	Case-control study	a
Getahun^[38]^	Ethiopia	2	19	Cross-sectional study	a
Ghugey^[39]^	India	7	375	Cross-sectional study	abc
Havas^[40]^	Georgia	60	49	Case-control study	a
Turhan^[41]^	Turkey	33	1087	Cross-sectional study	ab
Jackson^[42]^	Nepal	25	36	Cross-sectional study	a
Karagiannis^[43]^	Greece	98	5	Case-control study	b
Kiambi^[44]^	Kenya	60	386	Cross-sectional study	a
Lado^[45]^	South Sudan	58	116	Case-control study	a
Lita^[46]^	South Sudan	6	137	Cross-sectional study	ac
Majalija^[47]^	Uganda	15	185	Cross-sectional study	abf
Mehari^[48]^	Ethiopia	70	374	Cross-sectional study	a
Migisha^[49]^	Uganda	35	200	Cross-sectional study	ae
Obaidat^[50]^	Jordan	63	875	Cross-sectional study	a
Castell Monsalve^[51]^	Spain	74	58	Case-control study	a
Muturi^[52]^	Kenya	43	86	Case-control study	ae
Nasinyama^[53]^	Uganda	20	125	Cross-sectional study	a
Nawaz^[54]^	Pakistan	419	2014	Cross-sectional study	a
Nguna^[55]^	Uganda	19	426	Cross-sectional study	a
Onyango^[56]^	Kenya	96	84	Case-control study + Cross-sectional study	a
Mangtani^[57]^	India	40	1685	Cross-sectional study	ab
Rahman A^[58]^	Bangladesh	22	478	Cross-sectional study	a
Makala^[59]^	Tanzania	34	279	Cross-sectional study	acef
Ron-Román^[60]^	Ecuador	70	3663	Cross-sectional study	abe
Saddique^[61]^	Pakistan	45	401	Cross-sectional study	a
Al-Shamahy^[62]^	Yemen	235	234	Case-control study	ab
Sileshi^[63]^	Ethiopia	38	255	Cross-sectional study	a
Ullah^[64]^	Pakistan	33	200	Cross-sectional study	b
Yosef^[65]^	Somaliland	56	160	Cross-sectional study	acf

### 3.2. Meta-analysis of dietary risk factors

#### 3.2.1. Animal milk consumption (a)

**Figure 1.**
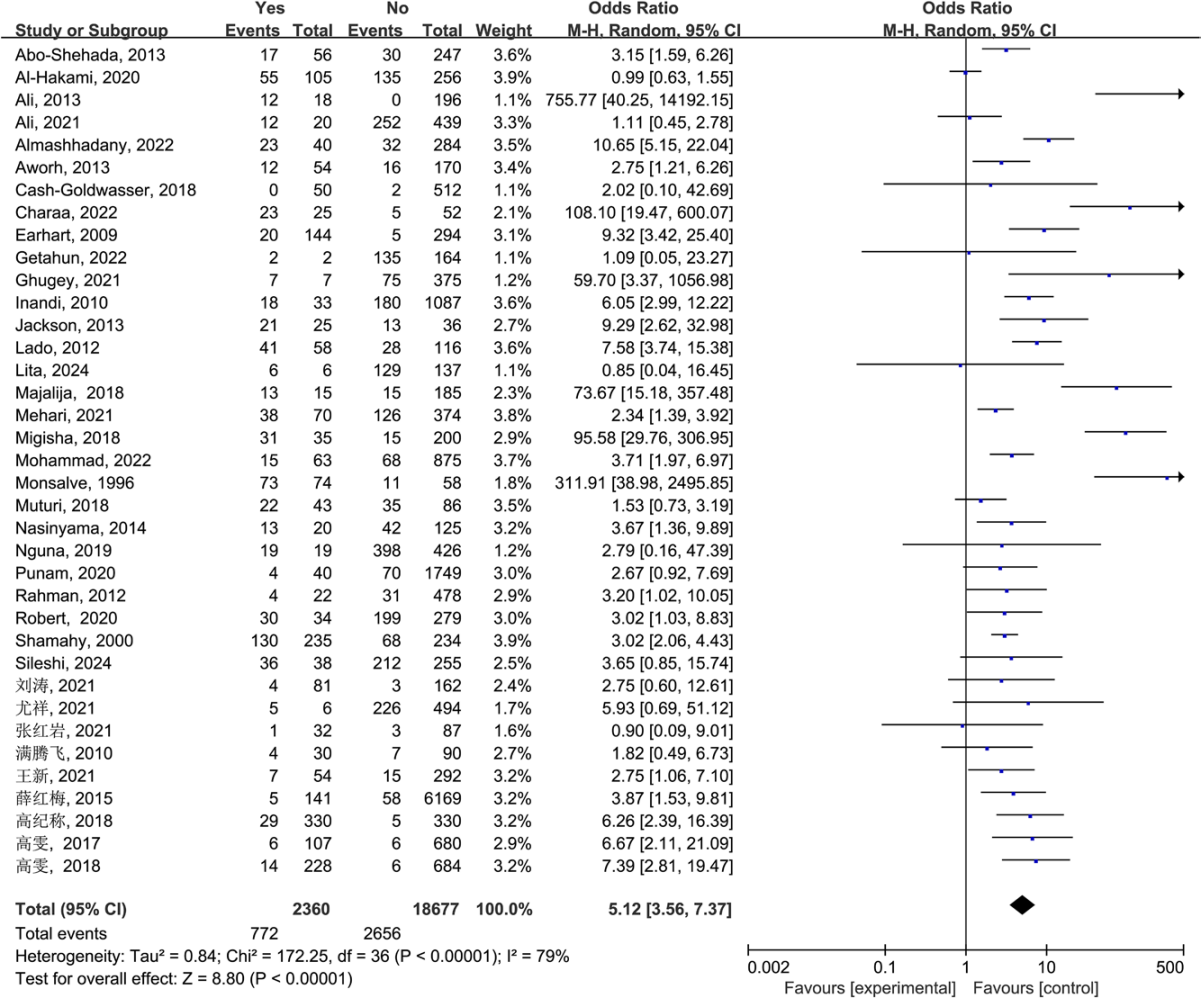
Forest plot of meta-analysis on the association between animal milk consumption and brucellosis incidence. CI = confidence interval.

In this study, 37 studies involving “animal milk consumption” as a risk factor were selected. Heterogeneity analysis showed that *I*^2^ = 79%, *P* < .00001, indicating significant heterogeneity, thus, a random-effects model were selected for pooling. The pooled effect size showed OR = 5.12, 95% CI: 3.56–7.37, which was statistically significant (*P* < .00001), suggesting that animal milk consumption is one of the risk factors for brucellosis (Fig. 1).

#### 3.2.2. Dairy consumption (b)

**Figure 2.**
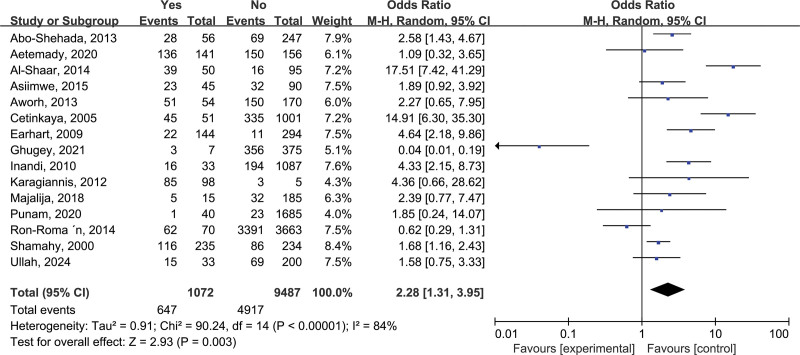
Forest plot of meta-analysis on the association between dairy consumption and brucellosis incidence. CI = confidence interval.

A total of 15 studies investigated “dairy consumption” were include in the final analysis heterogeneity analysis showed significant heterogeneity (*I*^2^ = 84%, *P* < .00001), so the random-effects model was used for pooling. After combining the effect size, the difference was OR = 2.28, 95% CI: 1.31–3.95, and the difference was statistically significant (*P* = .003, *P* < .005), that is, dairy consumption was one of the risk factors for brucellosis (Fig. 2).

#### 3.2.3. Raw or undercooked meat consumption (c)

**Figure 3.**
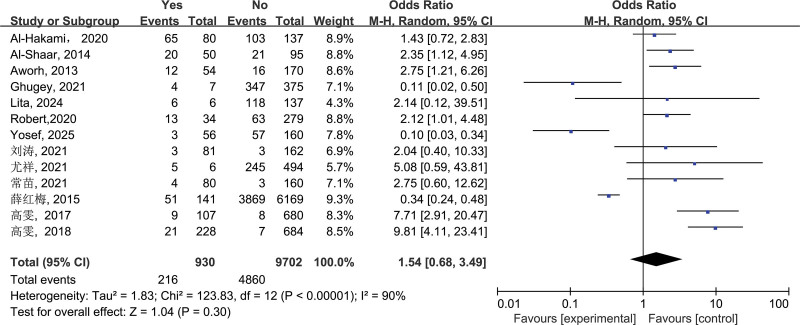
Forest plot of meta-analysis on the association between raw or undercooked meat consumption and brucellosis incidence. CI = confidence interval.

The final screening of this study included 13 studies on the relationship between “raw or undercooked meat consumption” and the incidence of brucellosis, which were tested to be significant (*I*^2^ = 90%, *P* < .00001), so the combined effect size was OR = 1.54, 95% CI: 0.68–3.49, and the difference was not statistically significant (*P* = .30, *P* > .05), that is, raw or undercooked meat consumption is not a risk factor for brucellosis transmission (Fig. 3).

#### 3.2.4. Sick or dead animal meat consumption (d)

**Figure 4.**
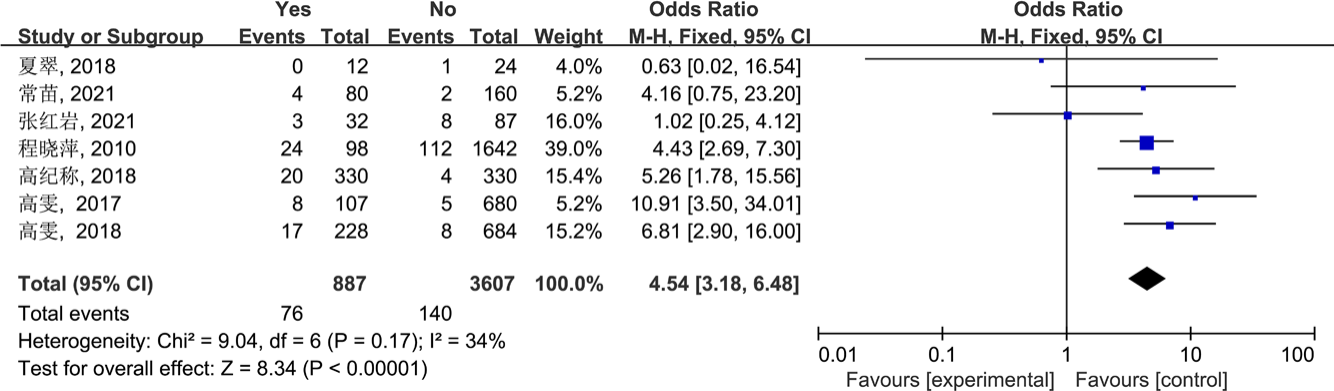
Forest plot of meta-analysis on the association between sick or dead animal meat consumption and brucellosis incidence. CI = confidence interval.

In the end, 7 studies were included in this study, all of which discussed “sick or dead animal meat consumption” as the risk factor of brucellosis. Subsequent heterogeneity test showed *I*^2^ = 34%, *P* = .17 (*P* > .05), suggesting that there was no significant heterogeneity among the studies. Therefore, a fixed-effect model was selected to combine effect sizes for combined analysis. The combined effect size OR = 4.54, 95% CI: 3.18–6.48, and the difference was statistically significant (*P* < .00001). Therefore, it can be concluded that one of the risk factors for brucellosis transmission is sick or dead animal meat consumption (Fig. 4).

#### 3.2.5. Blood consumption (e)

**Figure 5.**
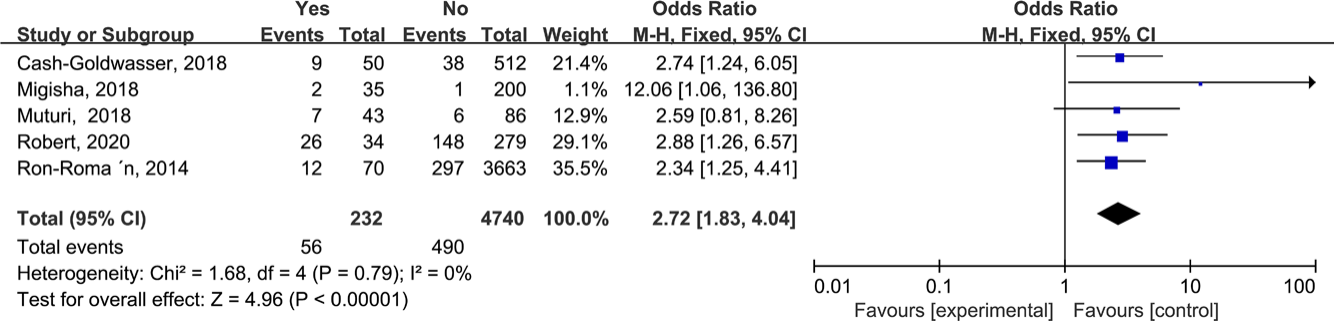
Forest plot of meta-analysis on the association between blood consumption and brucellosis incidence. CI = confidence interval.

A total of 5 studies were included in the final screening, including the relationship between “blood consumption” and brucellosis. Then, after heterogeneity test, the final results showed that *I*^2^ = 0%, *P* = .79 (*P* > .05), suggesting that the heterogeneity among various studies was low, so the fixed effects model was used for merging. After combining effect sizes, OR = 2.72, 95% CI: 1.83–4.04, *P* < .00001. The results showed that one of the risk factors for brucellosis transmission was blood consumption (Fig. 5).

#### 3.2.6. Zoonotic drinking water sources (f)

**Figure 6.**
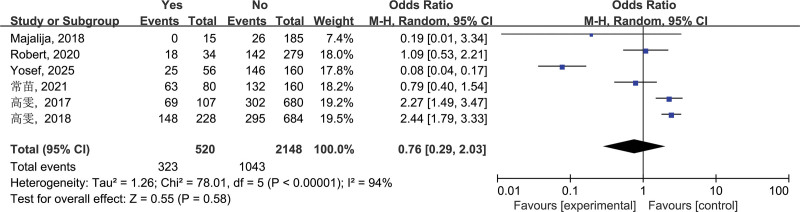
Forest plot of meta-analysis on the association between zoonotic drinking water sources and brucellosis incidence. CI = confidence interval.

According to the admission criteria, a total of 6 studies in the final screened literature involved the relationship between “zoonotic drinking water sources” and brucellosis. Subsequent heterogeneity test results showed *I*^2^ = 94%, *P* < .00001, suggesting significant heterogeneity among various studies, so random-effects model was used to merge. After the final combined effect size, OR = 0.76, 95% CI: 0.29–2.03, *P* = .58 (*P* > .05), the results showed that zoonotic drinking water sources were not risk factors for brucellosis transmission (Fig. 6).

#### 3.2.7. Poor hand hygiene before meals (g)

**Figure 7.**
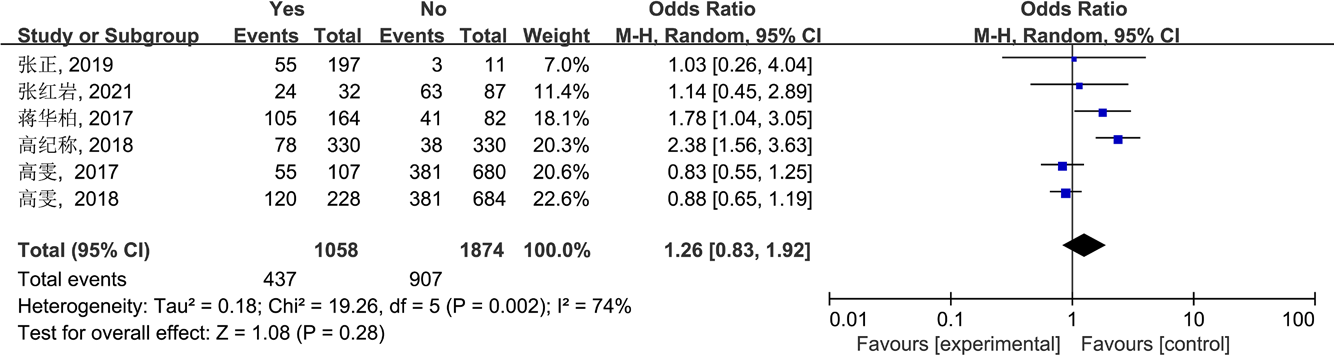
Forest plot of meta-analysis on the association between poor hand hygiene before meals and brucellosis incidence. CI = confidence interval.

A total of 6 studies included in the final screening literature used “poor hand hygiene before meals” as a risk factor. The results of heterogeneity test showed that *I*^2^ = 74%, *P* = .002 (*P* < .05), suggesting that there was significant heterogeneity among studies, so the random-effects model was used to combine the effect sizes. After pooling, OR = 1.26, 95% CI: 0.83–1.92, the difference was not statistically significant (*P* = .28, *P* > .05). These results indicate that poor hand hygiene before meals is not significantly associated with brucellosis transmission and thus is not a risk factor for the disease (Fig. 7).

### 3.3. Publication bias and sensitivity analysis

**Figure 8.**
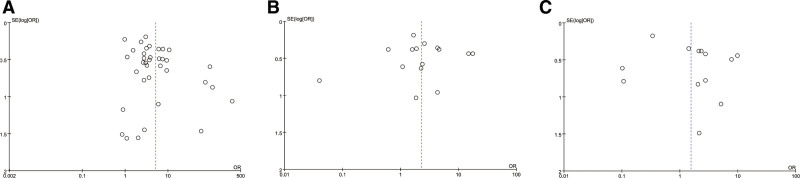
Funnel plot of the association between milk consumption (A), dairy consumption (B), raw or undercooked meat consumption (C), and brucellosis incidence.

**Figure 9.**
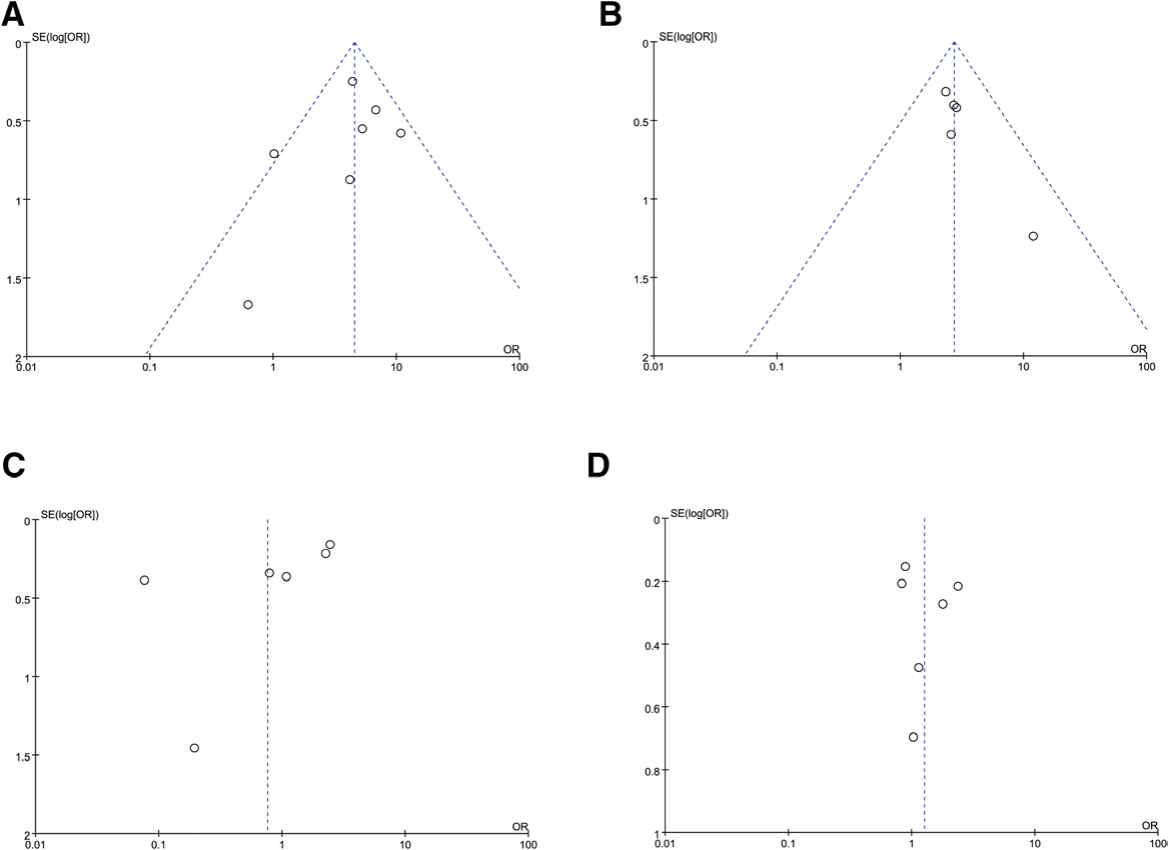
Funnel plot of the association between consumption of sick and dead animal meat(A), blood consumption(B), zoonotic drinking water sources(C), poor hand hygiene before meals (D), and brucellosis incidence.

Publication bias in this meta-analysis was evaluated by generating funnel plots for each dietary factor (Table 2). In order to detect publication bias, scatter plots with effect size *X*-axis and sample size *Y*-axis can be constructed, and whether the scatter points exhibit a symmetrical inverted funnel shape under fixed effects model, while whether the scatter points are evenly distributed on both sides of the regression line can be observed under random-effects model. The funnel diagrams tested for each dietary factor are basically funnel symmetrical or left-right symmetrical (Figs. 8 and 9). It shows that there is no obvious publication deviation of the 57 studies selected by us, which provides supplementary evidence for the reliability of the research results. Among the dietary risk factors, there is no heterogeneity in the studies of eating dead livestock meat and drinking blood. The research team conducted sensitivity analysis on these 2 association studies (Figs. S1 and S2, Supplemental Digital Content, https://links.lww.com/MD/R417). The results show that the comparative analysis between the fixed-effect model and the random-effect model shows that the combined results of effect values are consistent, suggesting that the interference degree of the low sample size study on the overall effect is in an acceptable range, which further strengthens the credibility of the research conclusion.

**Table 2:** Quality assessment for included studies.

Included studies	Study comparability	Comparability of groups	Outcome	Total
Chang^[9]^	3	2	3	8
Cheng^[10]^	3	1	2	6
Gao^[11]^	3	1	2	6
Gao^[12]^	3	1	2	6
Gao^[13]^	3	1	2	6
Jiang^[14]^	4	1	2	7
Liu^[15]^	4	1	2	7
Man^[16]^	4	1	2	7
Wang^[17]^	3	1	2	6
Xia^[18]^	3	1	2	6
Xue^[19]^	3	1	2	6
You^[20]^	3	2	3	8
Zhang^[21]^	4	1	2	7
Zhang^[22]^	3	2	3	8
Abbas^[23]^	4	1	2	7
Abo-Shehada^[24]^	4	1	3	8
Etemadi^[25]^	4	1	3	8
Al-Hakami^[26]^	4	1	3	8
Ali^[27]^	3	1	3	7
Ali^[28]^	3	1	2	6
Almashhadany^[29]^	3	1	3	7
Al-Shaar^[30]^	4	2	3	9
Asiimwe^[31]^	3	1	2	6
Aworh^[32]^	3	1	2	6
Cash-Goldwasser^[33]^	4	1	3	8
Cetinkaya^[34]^	4	1	3	8
Charaa^[35]^	3	1	3	7
Earhart^[36]^	4	1	3	8
Ejaz^[37]^	3	1	3	7
Getahun^[38]^	4	1	2	7
Ghugey^[39]^	4	1	3	8
Havas^[40]^	4	1	2	7
Turhan^[41]^	4	1	3	8
Jackson^[42]^	3	1	2	6
Karagiannis^[43]^	4	1	2	7
Kiambi^[44]^	3	1	3	7
Lado^[45]^	4	1	3	8
Lita^[46]^	3	1	3	7
Majalija^[47]^	4	1	3	8
Mehari^[48]^	4	1	3	8
Migisha^[49]^	3	1	2	6
Obaidat^[50]^	4	1	3	8
Castell Monsalve^[51]^	3	1	2	6
Muturi^[52]^	4	1	3	8
Nasinyama^[53]^	3	1	2	6
Nawaz^[54]^	4	1	3	8
Nguna^[55]^	4	2	3	9
Onyango^[56]^	4	1	3	8
Mangtani^[57]^	4	1	3	8
Rahman A^[58]^	3	1	2	6
Makala^[59]^	3	2	3	8
Ron-Román^[60]^	3	1	2	6
Saddique^[61]^	3	1	2	6
Al-Shamahy^[62]^	3	1	3	7
Sileshi^[63]^	4	1	3	8
Ullah^[64]^	4	1	2	7
Yosef^[65]^	4	1	3	8

## 4. Discussion

Brucellosis, also known as undulant fever, is a globally prevalent zoonotic disease that poses a severe threat to public health and the livestock industry.^[66]^ Brucellosis has a wide range of hosts, including cattle, sheep, pigs and other livestock. These animals often have hidden symptoms after infection and are difficult to detect at an early stage. Humans contract brucellosis mainly through ingestion of unsterilized dairy products or direct contact with brucellosis-infected animals.^[67]^ Incubation period of brucellosis usually lasts for 1 to 4 weeks. After infection, the main manifestations are chills, fever, hyperhidrosis, fatigue, muscle and joint pain, etc, and it presents “wavy fever.” As the disease progresses, brucellosis can further affect the nervous system, urinary system and reproductive system, causing serious health effects to patients.^[68-70]^

The routes of transmission of brucellosis mainly include dietary habits and direct contact with infected animals. Through meta-analysis, we found that drinking animal milk, dairy products, meat of sick and dead livestock, and blood were the main risk factors of brucellosis in eating habits. Although people’s awareness of food safety is gradually improving, in some epidemic areas, residents are not aware of the harm of brucellosis, and they still keep the habit of drinking raw milk and semi-raw meat.^[71]^ From the perspective of pathogen transmission route, unpasteurized raw milk, and dairy products may carry Brucella. After humans drink or eat it, the germs can enter the human body through the digestive tract and cause infection. However, the meat of dead animals and the blood of diseased animals often contain a lot of Brucella. If you eat these undercooked animal products, the germs will enter the human body with food, increasing the risk of infection.^[72]^

This study found that animal milk consumption, dairy consumption, sick or dead animal meat consumption and blood consumption are the main risk factors of brucellosis in eating habits. First of all, animal milk consumption is one of the important ways to spread brucellosis. Raw milk that has not been pasteurized may contain Brucella, and humans are susceptible to infection after consumption. Secondly, dairy consumption is also one of the ways of brucellosis transmission. Dairy products that are not sufficiently sterilized may contain Brucella, which will also increase the risk of infection after consumption. In addition, sick or dead animal meat consumption and blood consumption are also important ways to spread brucellosis. The meat of dead animals and the blood of sick animals often contain a large amount of Brucella. If you eat these undercooked animal products, the germs will enter the human body with food, increasing the risk of infection. In addition, the other relevant factors were not identified as risk factors for brucellosis, potentially attributable to the following reasons. First, Brucella has weak environmental resistance and has a short survival time in water sources and on frequently touched surfaces. Consequently, its transmission efficiency via drinking water or hand contact is substantially lower than that through the ingestion of contaminated animal-derived food via the digestive tract. Second, most relevant studies have been conducted in non-endemic areas, which fail to reflect the actual infection risk profile of exposed populations in endemic regions. Third, the included literature on the aforementioned factors is characterized by small sample sizes, and some studies have methodological flaws (e.g., failure to strictly control confounding factors). These limitations result in a lack of statistically significant correlations in the analyses, precluding the establishment of a robust evidence chain for causal inference. According to the risk factors of brucellosis dietary habits found in this study, comprehensive prevention and control strategies can be adopted. First of all, advocate not drinking raw milk and unsterilized dairy products. Strengthening quality supervision and quarantine of the production and circulation of dairy products, the safety of dairy products can be ensured. Secondly, it is advocated not to eat sick and dead livestock meat and cook the meat thoroughly. Strengthening quality supervision and quarantine of meat production and circulation, the safety of meat can be ensured. In addition, abandoning the custom of drinking blood is also one of the important measures to prevent and control brucellosis.^[73]^ Popularizing the prevention and control knowledge of “3 noes” (no raw milk, no dead livestock meat, no blood) to the public through multiple channels can effectively reduce the risk of brucellosis infection through diet. Raising public awareness of zoonotic diseases such as brucellosis is essential to prevent their spread and control outbreaks. Popularizing the knowledge of brucellosis transmission routes, symptoms and preventive measures to the public through multiple channels can effectively improve the public’s awareness of self-protection. For example, the knowledge about brucellosis can be publicized to the public through various media channels such as television, radio and the Internet. In addition, the knowledge of brucellosis prevention and control can be popularized to the public by holding health lectures and distributing publicity materials.

Although this study has achieved some results on risk factors of brucellosis dietary habits, there are still some limitations. First, subgroup analysis was not performed for certain risk factors (e.g., animal milk consumption) to assess the specific impact of different milk types (e.g., cow’s milk, goat’s milk, camel’s milk) on brucellosis infection risk. Secondly, the number of studies included in some dietary risk factors is insufficient, which may lead to the possibility of publication bias, thus affecting the final research conclusion. Future studies can further expand the sample size and carry out more in-depth subgroup analysis to gain a more comprehensive understanding of brucellosis transmission routes and prevention and control strategies.

## 5. Conclusion

Animal milk consumption, dairy consumption, sick and dead animal meat consumption, and blood consumption are the main risk factors of brucellosis. Promoting the adoption of healthy dietary habits may help reduce the risk of brucellosis infection.

## Author contributions

**Conceptualization:** Lei Feng.

**Data curation:** Lei Feng, Yi Shang.

**Funding acquisition:** Lei Feng, Yi Shang.

**Methodology:** Yi Shang.

**Project administration:** Yi Shang.

**Resources:** Lei Feng, Yi Shang.

**Validation:** Lei Feng.

**Writing – original draft:** Lei Feng.

**Writing – review & editing:** Lei Feng.

## Supplementary Material


