# Supplementary material for: Meta-analysis of brucellosis and relative risks
Source: Medicine (Baltimore). 2026 Feb 28;105(9):e47696. doi: 10.1097/MD.0000000000047696 (PMC12956228; doi:10.1097/MD.0000000000047696)

Supplementary Figures

Fig.S1 Sensitivity analysis of sick and dead animal meat consumption and brucellosis incidence


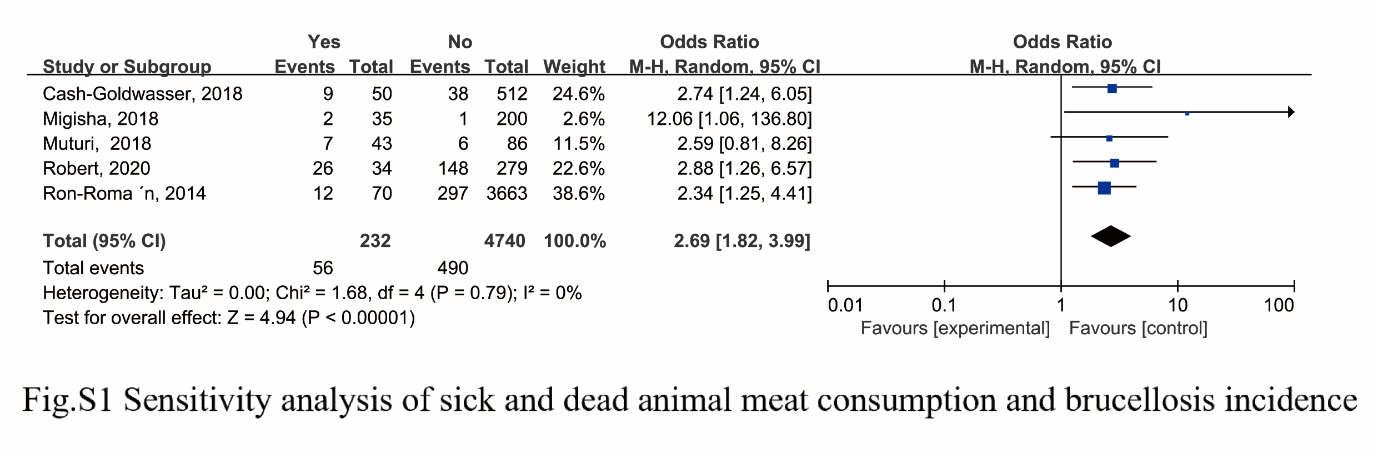


Fig.S2 Sensitivity analysis of blood consumption and brucellosis incidence


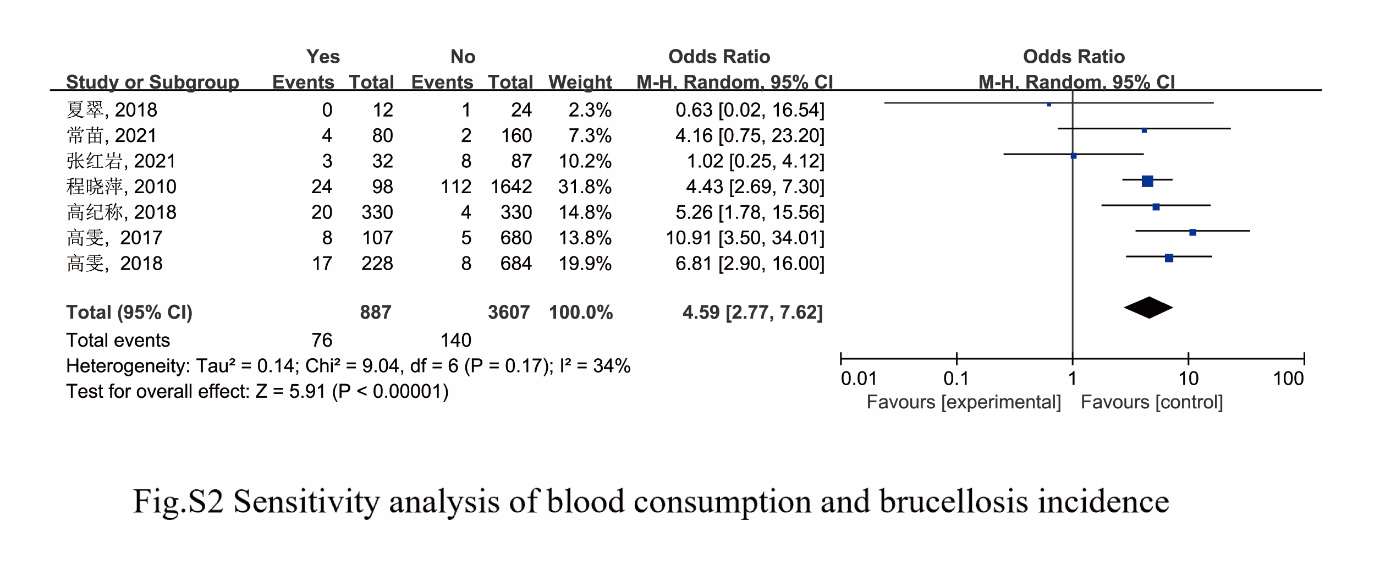

Supplement: Supplementary file 1 [file medi-105-e47696-s001.docx]
